# Why Are There so Many Plant Species That Transiently Flush Young Leaves Red in the Tropics?

**DOI:** 10.3389/fpls.2020.00083

**Published:** 2020-02-18

**Authors:** Wei-Chang Gong, Yan-Hong Liu, Chuan-Ming Wang, Ya-Qing Chen, Konrad Martin, Ling-Zeng Meng

**Affiliations:** ^1^ College of Life Science & Technology, Honghe University, Mengzi, China; ^2^ Institute of Agricultural Sciences in the Tropics, Hans-Ruthenberg-Institute, University of Hohenheim, Stuttgart, Germany; ^3^ Xishuangbanna Tropical Botanical Garden, The Chinese Academy of Sciences, Mengla, China

**Keywords:** red coloration, young leaves, tropics, plant defense, anthocyanins, tannins

## Abstract

Delayed greening of young leaves is a ubiquitous and visually striking phenomenon in the tropics. Here, we investigated the potential ecological functions of red coloration patterns in young leaves. To detect any protective function of the red coloration on the young leaves, leaf damage by insect herbivores was recorded in the field. To determine capacity for chemical defense, the concentrations of tannins and anthocyanins were measured in both young and mature leaves. To test the hypothesis that anthocyanins function as photo-protective molecules, chlorophyll content, maximum photochemical efficiency of *PSII* (*F* *_v_*/*F_m_*), non-photochemical quenching (*NPQ*), and effective quantum yield of *PSII* (*Φ_PSII_*) were measured in the field. Phylogenetic relationships were analyzed to test the relationary significance of the occurrence of redness in young leaves. Compared to the coloration in non-red leaves, young red leaves had significant higher anthocyanins and tannins content and lower herbivore damages. Young, red leaves had the lowest *F_v_*/*F_m_* values, which were significantly lower than those of non-red leaves. *NPQ* values in young red leaves were comparable to those of other groups. Although young red leaves had high *Φ_PSII_*, these values were significantly lower than those of the other three groups. The results suggest that the red coloration of young leaves protects them from insect herbivory primary by chemical defense through high concentrations of tannins and anthocyanins. Additionally, low *F_v_*/*F_m_* values in young red leaves indicate that anthocyanins might not be functioning as light attenuators to compensate for insufficient photo-protection mediated by *NPQ*. And finally, red coloration in young leaves is predominantly a result of adaptation to heavy herbivory stress but without significant intrinsic phylogenetic relationship of plant species.

## Introduction

Plant species are sensitive to changes in environmental stimuli and shift their physiological development to adapt to their specific surroundings (Niu et al., 2014). Environmental changes can alter the availability of resources and other conditions crucial to plant performance (Nicotra et al., 2010). Plant leaves are not only the most important photosynthetic organs but also the parts of the plant that can most sensitively respond to environmental changes (Nicotra et al., 2010; Niu et al., 2014). In the field, leaf size, shape, thickness, and folding behavior (Parkhurst and Loucks, 1972; Givnish, 1987; Turner, 1994; Smith et al., 1997; Beerling et al., 2001; Cornelissen et al., 2003; Huang et al., 2012), and even secondary metabolites or leaf coloration (Coley and Aide, 1989; Gould et al., 1995; Westoby, 1998; Nicotra et al., 2010; Chen and Huang, 2013; Niu et al., 2014; Tellez et al., 2016; Wang et al., 2016) can all be altered to respond to abiotic environmental changes (e.g., UV light) or biotic herbivory stresses.

In the tropics, certain plant species have evolved species-specific leaf development patterns, such as delayed greening (Kursar and Coley, 1992). Young leaves in tropical regions worldwide often flush red synchronously during expansion (Dominy et al., 2002; Manetas et al., 2003). Red flushing can be a widespread and visually striking phenomenon in the tropics, with between 20%–40% of the woody species showing red flushed young leaves at a single site (Opler et al., 1980). Coley and Barone (1996) reported that approximately one third of the plant species in tropical forests delayed greening of their young leaves until full expansion. Kursar and Coley (1992) found that 36% of tree species exhibited red coloration of young leaves at Barro Colorado Island (BCI), Panama. Furthermore, Chapman et al. (1999) reported that nearly 49% of forest trees in Kibale National Park, Uganda exhibited redness of young leaves at some point over the years. The delayed greening phenomenon in tropical rainforests of Southeast Asia included as much as 62% of the total studied plant species (Dominy, 2002).

Young red leaves generally contain high levels of anthocyanins, which are the primary contributors to the red coloration of plant leaves (Field et al., 2001; Trojak and Skowron, 2017; Cooney et al., 2018). Anthocyanins are produced not only in response to seasonal, spatial or developmental factors but can also be induced by a number of disparate abiotic (e.g., ultraviolet (UV) light) and biotic (e.g., insect herbivores, folivorous mammals, pathogens) stimuli (Hammerschmidt, 1977; Hipskind et al., 1996; Merzlyak and Chivkunova, 2000; Gould et al., 2002a; Trojak and Skowron, 2017). During leaf expansion, anthocyanins are frequently present in mesophyll and epidermal cells and then disappear from the tissues or decrease in concentration after full leaf expansion (Trojak and Skowron, 2017). In plants, anthocyanins function to screen against harmful reactive oxygen species (ROS), herbivore attacks, fungal attacks, and high light stress (Coley and Aide, 1989; Gould et al., 1995; Gould et al., 2002b; Tellez et al., 2016). However, anthocyanins do not serve as photosynthetic pigments (Trojak and Skowron, 2017). Since a primary function of plant leaves is photosynthesis, why do so many plant species not show an optimized pigment pool, (in terms of the absorption spectrum required for efficient light capture for photosynthesis) but instead accumulate high concentrations of anthocyanins and delay greening at the juvenile stage?

The ecological significance of delayed greening in young leaves has been disputed (Coley and Kursor, 1996). Coley and Aide (1989) found that leaf-cutter ants preferentially pick up leaves without anthocyanins. These authors then proposed the hypothesis that anthocyanins are associated with against invasions by leaf-attacking fungal pathogens. This view was shared by Tellez et al. (2016). Stone (1979) reported that the redness of young leaves may make them invisible to herbivores or may warn potential herbivores of the presence of toxic compounds. Other researchers have found that anthocyanins may function as light attenuators to compensate for insufficient photo-protection mediated by non-photochemical quenching (*NPQ*) (Demmig-Adams and Adams, 1992; Gould et al., 1995; Hughes et al., 2005; Zhu et al., 2018). However, these hypotheses of delayed greening in young leaves were put forward or verified based on studies considering only a few plant species, and whether these mechanisms have widespread adaptive significance for tropical plants is still unclear.

Plant-herbivore interactions constitute an important component of tropical rainforest biodiversity. Reciprocal selection has led to greater investment in defenses in tropical trees than in temperate plant species (Levin and York, 1978; Coley and Aide, 1989; Basset, 1994; Marquis and Braker, 1994). Although plant leaves are easily damaged by generalist or specialist herbivores (Cates, 1980; Coley and Aide, 1989; Broadway and Colvin, 1992; van Dam et al., 1995; Coley and Barone, 1996; Blüthgen and Metzner, 2007), young leaves, especially those with red phenotypes, can effectively decrease herbivore damage (Kursar and Coley, 1991; Karageorgou and Manetas, 2006). However, Chen and Huang (2013) reported that young red leaves have fewer mechanical physical defenses than do green leaves. Thus, the mechanism by which red coloration of young leaves helps them avoid harm from herbivores in the tropics is an important area of phenotypic study.

The tropics usually experience greater natural selection than temperate areas. The strong intensity of natural selection processes in tropical areas, which can be either continuous or intermittent processes consisting of exacerbations and remissions, makes phylogenetic relatedness of plant species decreased in tropical areas. Moreover, these ecological factors and their evolutionary relationships with plant species in tropical rainforests have resulted in an impressive variety of adaptations and interactions (Coley and Barone, 1996).

In the present study, we investigate the metabolism, photosynthetic activities and chemical defenses of leaves from 250 tropical plant species with either red or green young leaves. Our major aim is to investigate why there are so many plant species with transiently red young leaves in the tropics. Additionally, we attempt to highlight any ecological or evolutionary significance of delayed greening in young leaves of plants in the tropics. Our study involved systematic data collection in a tropical region of southwest China, through which we aim to answer the following questions:

1. Is red coloration in young leaves associated with the phylogenetic relationship of plant species?
2. Do young red leaves have better protection against herbivores than green leaves?
3. Does red coloration play a role in photosynthesis and photo-protection?

## Materials and Methods

### Study Sites and Leaf Damage Investigation

This work was carried out between January 2016 and March 2017 in Southern Yunnan, China, with most plant samples coming from Xishuangbanna Tropical Botanical Garden (21°41´N and 101°25´E, 570 m a.s.l.) and a small number of samples, used for field observation of natural leaf damage, from the botanic garden of Honghe University (23°21´N and 103°25´E, 1331 m a.s.l).

To differentiate the effect that the hydrolysable tannin content in plants had on food plant preference in insect herbivores, an extensive field investigation *in situ* into the feeding patterns of insect herbivores feeding on leaves between early and late stages of maturation were carried out. Our field survey involved 83 red-flushing woody species from 36 families (here called delayed-greening leaves, DGL) and 167 woody species without red young leaves (here called green leaves, GL) from 70 families. Prior to the experiment, three to five trees from each taxon were selected. Herbivore damage to young and mature leaves in each tree was assessed. For each plant, 10 branches from different parts of the tree were selected, and a total of approximately 200 young and mature leaves were assessed for herbivore damage. Irregularly shaped or incomplete leaves were recorded as damaged. The third and the fourth expanding leaves from the apex of each branch were treated as young leaves. The other leaves after the seventh from the apex of each branch were regarded as mature leaves.

### Anthocyanins and Chlorophyll Content

We then determined the relative content of anthocyanins and the total chlorophyll content in both mature and young leaves, in order to investigate potential differences in anthocyanin and chlorophyll content between young and mature leaves. From the 250 plant species used in the field investigation into herbivory damage, we selected a total of 210 plant species (66 families, 150 genera), including DGL plant taxa (124 taxa, 43 families, 89 genera) and GL taxa (86 taxa, 42 families, 74 genera) to measure their concentrations of both anthocyanins and chlorophyll.

### Determination of Concentration of Anthocyanins

The relative concentration of anthocyanins was determined following Christie et al. (1994) and Zhu et al. (2015) with minor modifications. Pulverized leaf blade tissue (1.0 g fresh weight) was homogenized in 12 ml of methanol containing 0.1 N HCL and maintained at 4°C for 4 h. Three repetitions were performed for each taxon. Particulates were removed by centrifugation at 1000 r/min for 10 min, and the absorption of the anthocyanin extracts was measured by a spectrophotometry (UV 5100B, Shanghai Metash Instrument Co., Ltd., Shanghai, China) at 530 nm. The relative concentration of anthocyanins was then calculated as 10×A_530_×B, where A is the measured absorption at 530 nm, and B is the dilution ratio of the experimental supernatant.

### Chlorophyll Determination

Chlorophyll was extracted by using N, N-dimethylformamide, following Niu et al. (2014). The appropriate leaf area (0.2 cm^2^, fresh) of material for each species was punched from fresh leaves (avoiding major leaf veins where possible) through a circular leaf disc puncher (ø = 5 mm). Six 0.2 cm^2^ leaf disks were collected from six different leaves for each taxon, weighed fresh (to within 0.1 mg) were used and were then immersed in N, N-dimethylformamide for one night (~12 h) in the dark. Three repetitions were performed for each taxon. The resulting solutions were then centrifuged at 1000 r/min for 10 min, the supernatant was collected and the absorption values were measured using ultraviolet-visible spectrophotometry at 664.5 and 647 nm. The concentration of chlorophyll was calculated as equivalents per fresh weight of the leaves following Inskeep and Bloom (1985) and Niu et al. (2014).

### Measurement of Total Tannin Content

Tannic acid is regarded as a major component of the total phenolics within the leaf. Total phenolic content was determined using the Folin-Ciocalteau method, following Singleton and Rossi (1965) and Greer et al. (2014). Pulverized leaf blade tissue (0.5 g freeze-dried weight) was homogenized in 10 ml of ethyl alcohol (60%) and then heated in a water bath (60°C) for 3 h. Samples were weighed ( ± 0.1 mg) to obtain their fresh weight (FW) before extraction. Three repetitions were completed for each taxon. Particulates were removed by centrifugation at 1000 r/min for 10 min and then filtered through a Buchner funnel. 1.0 ml of the solution was then combined with 60 ml of dH_2_O water, 5.0 ml of Folin-Ciocalteau reagent and 15 ml of sodium carbonate in a 100 ml volumetric flask. A phenolic concentration standard curve was generated with tannic acid (concentrations from 0 to 50 mg/L). To better fit the standard curve, leachates were diluted to 8× their original concentrations. Both standard and samples were analyzed in a spectrophotometer at 765 nm. Total phenols are therefore reported as milligrams of tannic acid (gallotannin, Sigma) equivalents per gram fresh tissue (mg TAE/g FW).

### Chlorophyll a Fluorescence Activity

Chlorophyll a fluorescence parameters of young and mature leaves were investigated using an Li-6400XT portable photosynthesis system (LiCOR Inc., USA) with an integrated leaf chamber fluorometer (LCF). For this experiment, of the total 250 plant species mentioned, 133 plant species were used, including 71 DGL species and 62 GL species. Ten leaves (five young and five mature leaves) from each taxon were used separately for the assessment of chlorophyll a fluorescence.

The maximum photochemical efficiency of *PSII* (*F_v_*/*F_m_*), which can be used to quantify photo-inhibition (Redondo-Gómez et al., 2006), was then calculated following Figueroa et al. (1997) and Lima et al. (2018). On the following day (9:00–11:00), after light adaptation for half an hour, chlorophyll a fluorescence traits of the same leaves were estimated with the Li-6400XT portable photosynthesis system. Minimum fluorescence (*F_0_*) was measured under a continuous measuring light (1200 µmol quanta m^-2^s^-1^), and then *F_m_* was measured following a pulse of actinic light of 5000 µmol m^-2^s^-1^. Maximum quantum yield of *PSII* was calculated as

$$F_{v}/F_{m}=(F_{m}-F_{0})/F_{m}$$

After 30 min to allow for dark adaptation, the chlorophyll fluorescence parameters *F_0_*, *F_m_*, and *F_s_* of each of the young and mature leaves were measured. *F_0_* is the minimum chlorophyll a fluorescence after dark adaptation; *F_m_* is the maximal fluorescence level in the dark-adapted state, and *F_s_* is the steady-state fluorescence yield (Lima et al., 2018).The effective quantum yield of *PSII* (*Φ_PSII_*), which can be used for routine assays of plant heath performance and the quantification of environmental stress, was calculated following Redondo-Gómez et al. (2006) and Lima et al. (2018):

$$\Phi_{\text{PSII}}=(F_{m^{\prime }}-F_{s})/F_{m^{\prime }}$$

Non-photochemical quenching (*NPQ*), which is an important photo-protective process in plants, was also calculated as follows (Redondo-Gómez et al., 2006; Ware et al., 2015; Lima et al., 2018):

$$\text{NPQ}=(F_{m}-F_{m^{\prime }})F_{m^{\prime }}$$

### Phylogenetic Relationships Between DGL and GL Taxa

The family and genus names of all the studied species (210 species in total) in the APG III system were obtained using the R package plantlist (Zhang, 2018). The phylogenetic relationships between these species were examined using the online tool Phylomatic (Webb and Donoghue, 2005) (www.phylodiversity.net/phylomatic/) based on the Angiosperm consensus tree from Davies et al. (2004). The results were visualized uisng the iTOL (itol.embl.de) online tool for the display and annotation of phylogenetic trees (Letunic and Bork, 2006; Letunic and Bork, 2016). A total of 204 species were shown in the tree, and six species (*Balakata baccata*, *Lagerstroemia siamica*, *Lithocarpus microspermus*, *Nyssa sinensis*, *Parakmeria yunnanensis*, *Stixis suaveolens*) were not because of no record in database. Branch lines and the background of the terminal nodes were colored, displaying DGL and GL plant species. Ten different color strings outside of the tree illustrated the family subtrees, including more than five species. The numerators and denominators of the fractions behind the family names give the number of DGL species and the total number of species studied in this family, respectively.

### Statistical Analyses

For each measured parameter, the mean values for each species were used for further calculation and for presentation in figures. A list of all the plant species used in the measurement of photosynthesis physiology or determination of defense chemical content is supplied in the supporting information (**Table S1**). Nonparametric Kruskal–Wallis ANOVAs were conducted to test for differences in the percentage variation of leaf area damaged by insect herbivores between the young red and mature green leaves. GL plant species were compared in the same way. The effects of the color of the young leaf and species on the chlorophyll fluorescence parameter which shown the photosynthetic ability and potential protection mechanism, including *F*_v_/*F*_m_, effective *PSII* efficiency, and non-photochemical dissipation (*NPQ*) were evaluated using two-way analysis of variance (ANOVA). Differences in anthocyanin, chlorophyll and tannin content between young and mature leaves were assessed using an *F* test after all sample data had been tested for normality. A general Pearson linear regression and a Spearman's Rho non-parametric correlation were used to examine any potential relationships between the contents of anthocyanin and tannin in leaves, respectively. All of the statistical analyses were performed with the SPSS v.16.0 statistical package (SPSS, Chicago, IL). As well as the phylogenetic correlation analysis described above, we used two-tailed *U*-tests to compare the proportions of DGL and GL plant species in each family of the phylogenetic branch, to test the null hypothesis that of the color of the young leaves was not associated with their phylogenetic placement. The numbers present and absent in green species were set as the expected numbers. Figures were generated using SigmaPlot software (Version 14.0, Systat Software, San Jose, CA).

## Results

### Field Investigation of Leaf Damage

**Figure 1:**
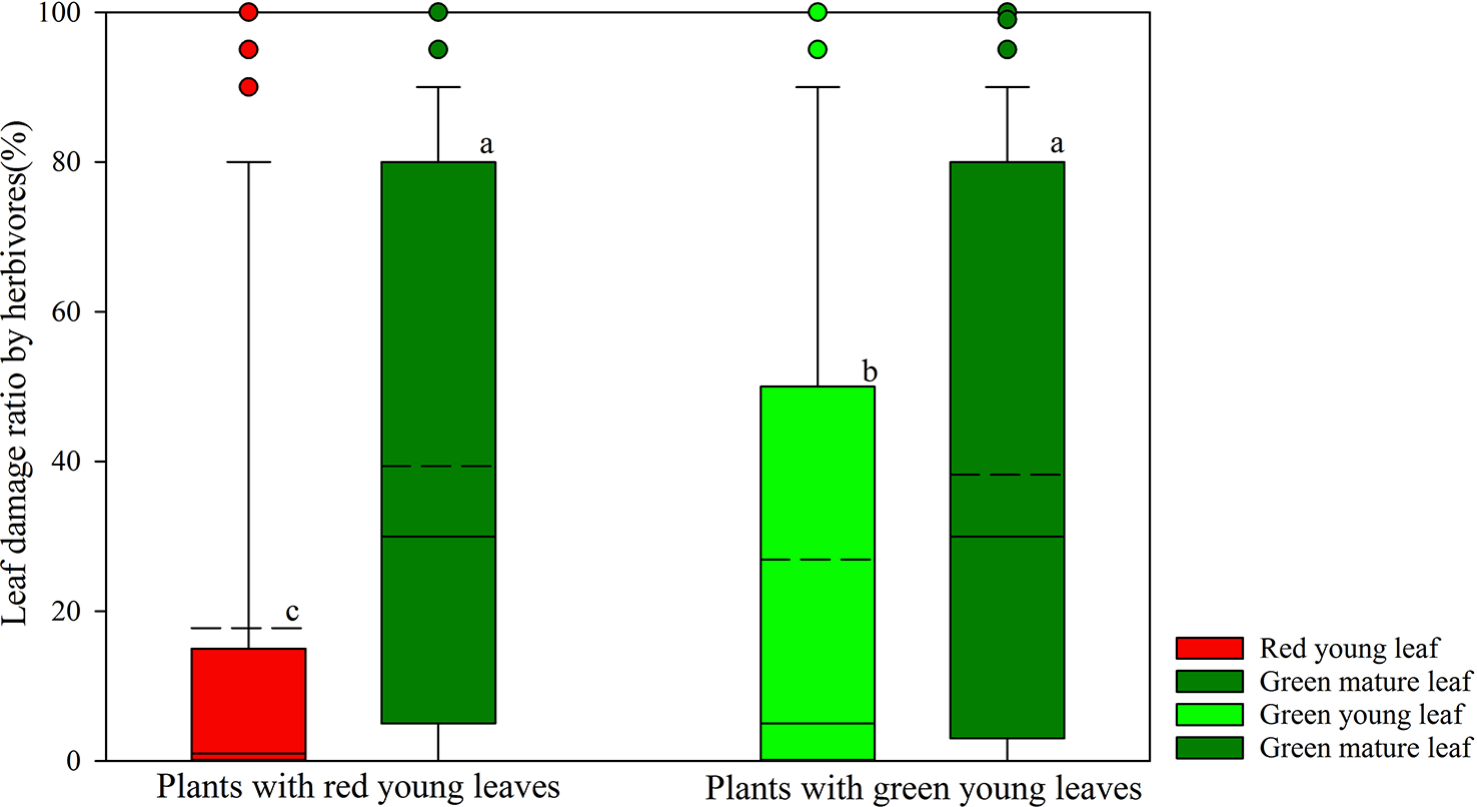
Differences of leaf damage by herbivores in young and mature leaves in red- and green-young leaf taxa. Box and whisker plots illustrate the 5th, 25th, 50th (median), 75th, and 95th percentiles, and the means are given as a short dashed line. Different letters indicate significant differences (*P* < 0.05; Nonparametric Kruskal–Wallis ANOVAs test). Black small circles represent outliers.

Herbivore damage to young and mature leaves of plant species was investigated in the field (**Figure 1**). Young leaves of both DGL and GL species showed significantly lower herbivore damage proportions than did the mature leaves on the same plants (*P* < 0.001; *P* < 0.001, total n = 250). The young GL leaves were more attacked by insect herbivores (26.89 ± 2.72%, n = 167) than young DGL leaves (17.75 ± 3.30%, n = 83) (*P* < 0.01). Approximately 39% (n = 83) of the green mature leaves from DGL plant species were damaged by herbivores, and 38% (n = 167) of the green mature leaves of GL plant species were damaged. There was no significant difference between DGL and GL groups (*P* > 0.05) (**Figure 1**).

### Leaf Chlorophyll, Anthocyanin and Tannin Characteristics

**Figure 2:**
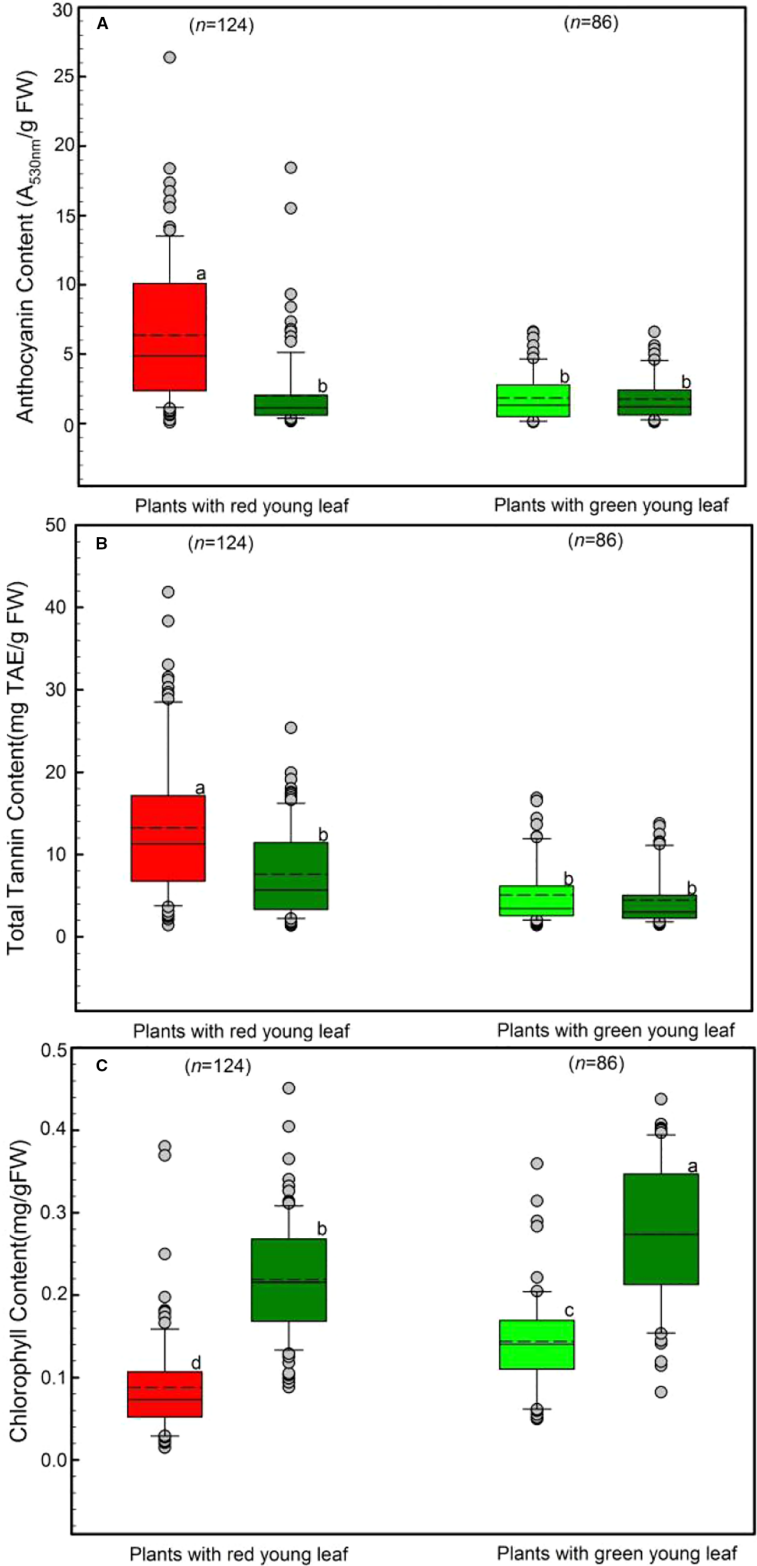
Differences in leaf anthocyanin **(A)**, tannin **(B),** and chlorophyll **(C)** concentrations of young and mature leaves in red- and green-young leaf taxa. Box and whisker plots illustrate the 5th, 25th, 50th (median), 75th, and 95th percentiles, and the means are given as a short dashed line. Different letters indicate significant differences (*P* < 0.05; *F*-test). Grey small circle represent outliers.

We determined the levels of anthocyanins, tannin and chlorophyll characteristics in young and mature leaves (**Figure 2**). Both young leaves from DGL (n = 124) and from GL (n = 86) were investigated.

Anthocyanins in young and mature leaves were also evaluated, as shown in **Figure 2A**. Young leaves with red coloration had the greatest anthocyanin concentration, Around 6.45 ± 0.66 A_530nm_/g FW anthocyanins were detected in young DGL leaves. This value was significantly higher than those from the other three groups (*F*_3,__417_ = 38.78, *P* < 0.001). In contrast, both young and mature GL leaves had relatively low levels of anthocyanins, ranging from 1.76 ± 0.23 to 2.14 ± 0.38 A_530nm_/g FW (**Figure 2A**). There were no significant differences between these three groups (*P* > 0.05).

The tannin content of young and mature leaves is presented in **Figure 2B**, and shows the same trend as the leaf anthocyanin (**Figure 2A**). The highest tannin content was observed in young DGL leaves (13.81 mg TAE/g FW), which was significantly greater than values from the three non-red groups (*F*_3,__417_ = 34.6, *P* < 0.001). The mature leaves from DGL taxa showed the second highest level of tannin (7.86 mg TAE/g FW) (**Figure 2B**). The leaf tannin concentrations in GL taxa were significantly lower than those in DGL taxa. No significant differences between young and mature leaves were found (*t*-test, *P* > 0.05).

The total chlorophyll content varied significantly between the four groups (*P* < 0.001; **Figure 2C**). Mature leaves from all studied species had greater total chlorophyll content than did young leaves from the same species (**Figure 2C**). The total chlorophyll content was 0.14 mg/g FW in GL taxa. Moreover, mature leaves of DGL plant taxa contained more chlorophyll than young leaves from those two groups (*P* < 0.01). Additionally, the young DGL leaves had the lowest chlorophyll content, 0.09 mg/g (**Figure 2C**).

**Figure 3:**
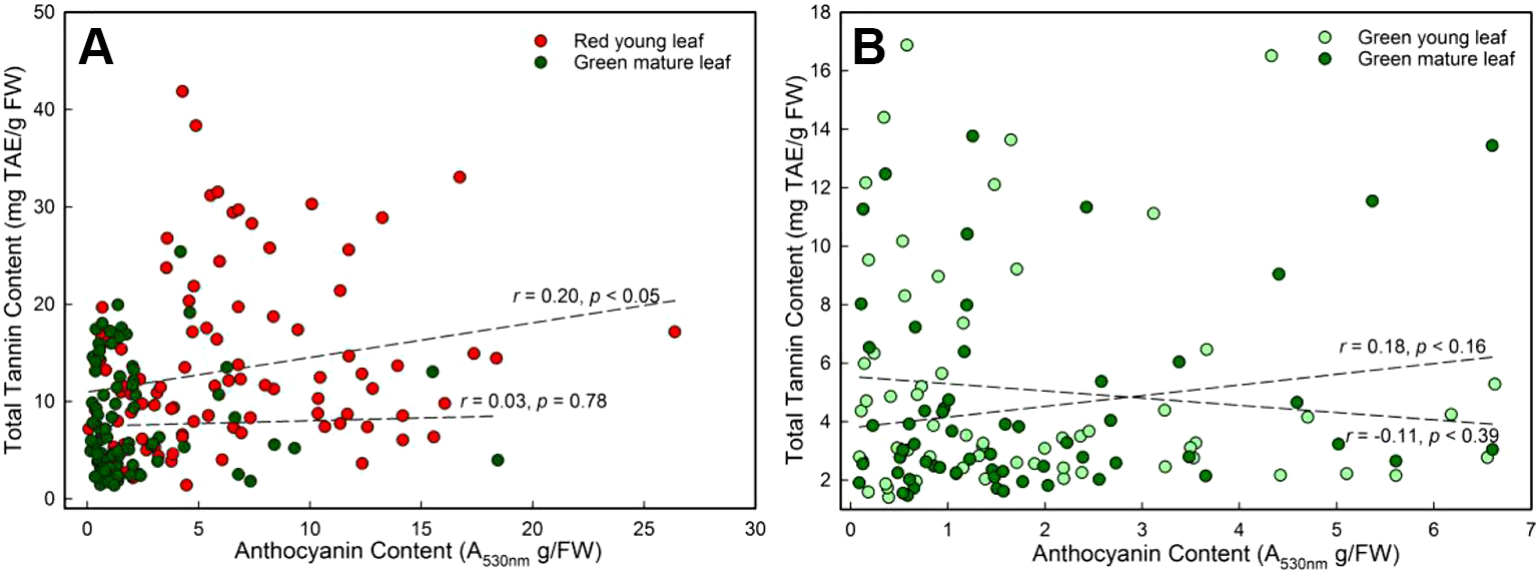
The general Pearson linear correlation between leaf anthocyanin and tannin contents of young and mature leaves in red- and green-young leaf taxa. **(A)** red-young leaf taxa (n = 96); **(B)** green-young leaf taxa (n = 61).

A significant correlation between anthocyanin and tannin contents of young DGL leaves was found (*r* = 0.20, *P* < 0.05, Pearson linear correlation; *r* = 0.30, *P* < 0.01, Spearman's *rs* correlation; n = 96, **Figure 3A**). However, this relationship was not observed in any of the other three groups (*P* > 0.05, **Figure 3**).

### Chlorophyll Fluorescence Activity

**Figure 4:**
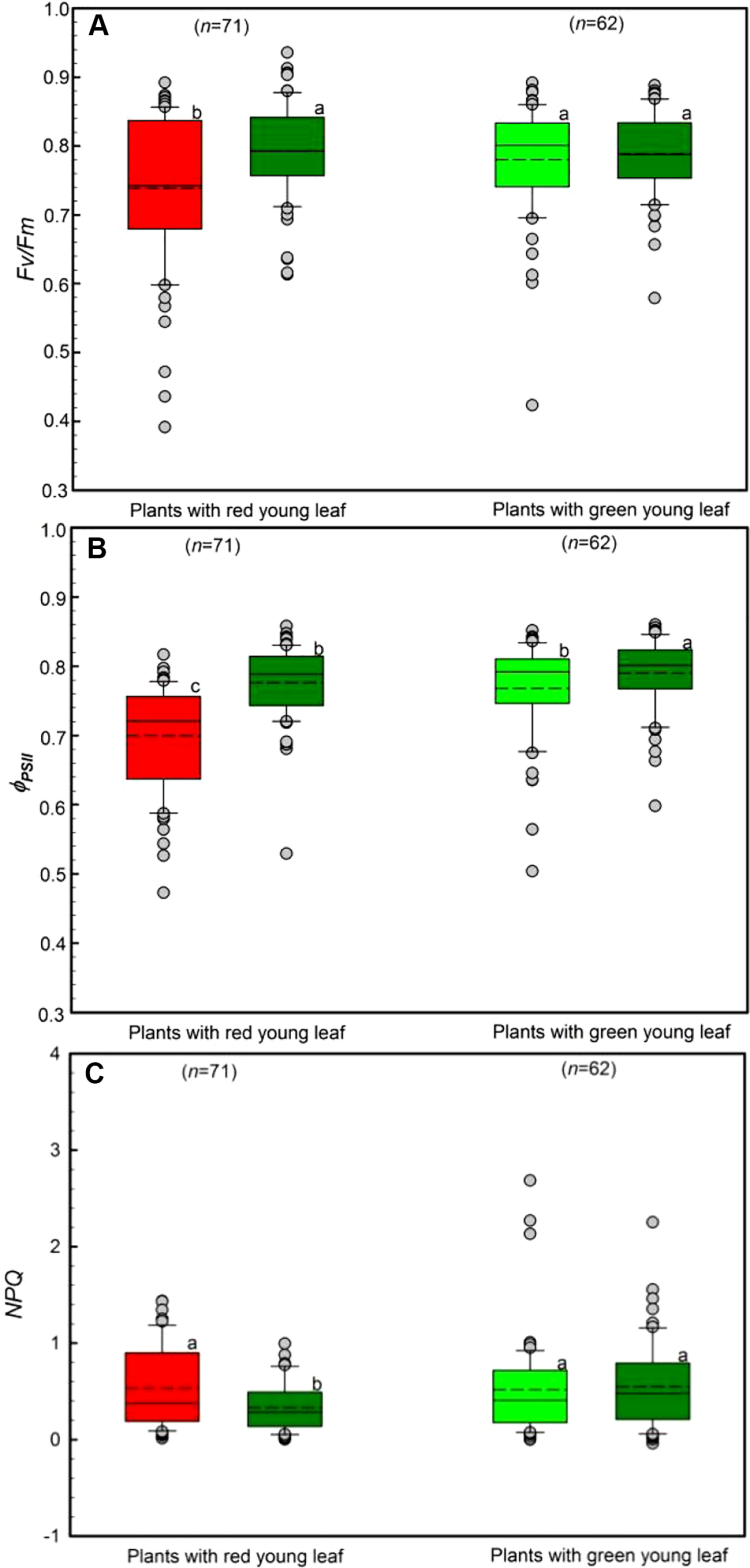
Chlorophyll a fluorescence parameters including *F_v_*/*F_m_* **(A)**, *Φ_PSII_* **(B)** and *NPQ* **(C)** of young and mature leaves in red- and green-young leaf taxa. Box and whisker plots illustrate the 5th, 25th, 50th (median), 75th, and 95th percentiles, and the means are given as a short dashed line. Different letters indicate significant differences (*P* < 0.05; two way ANOVAs test). Grey small circle represent outliers.

The chlorophyll a fluorescence of young and mature leaves was evaluated in the field (**Figure 4**). Young leaves from DGL (n = 71) and GL (n = 62) species were used. Both young and mature GL leaves had high *F_v_*/*F_m_* values, ranging from 0.74 to 0.79 (**Figure 4A**). Young DGL leaves had the lowest *F_v_*/*F_m_* values, 0.74, which were significantly lower than the values of the other three non-red leaf groups (*P* < 0.001). There were significant differences (*P* < 0.05) between young and mature leaves in GL leaf taxa.

The differences in *Φ_PSII_*, the quantum efficiency of *PSII* photochemistry, between young and mature leaves was assessed (**Figure 4B**). All studied taxa showed relatively high *Φ_PSII_* values, ranging from 0.70 to 0.78. Young DGL leaves exhibited the lowest *Φ_PSII_* values, which were significantly different from the other three non-red groups (*P* < 0.001). A significant difference among GL leaf taxa was found (*P* < 0.05).

Differences in the *NPQ* values between young and mature leaves were evaluated, ranging from 0.48 to 0.59 (**Figure 4C**). There were significant differences among the four groups (*P* < 0.05). In red-young leaf taxa, *NPQ* values in young red leaves were significant greater than in their mature leaves (*P* < 0.05). However, there were no significant differences between red- and green-young leaves (*P* > 0.05) (**Figure 4C**).

### Phylogenetic Reconstruction

**Figure 5:**
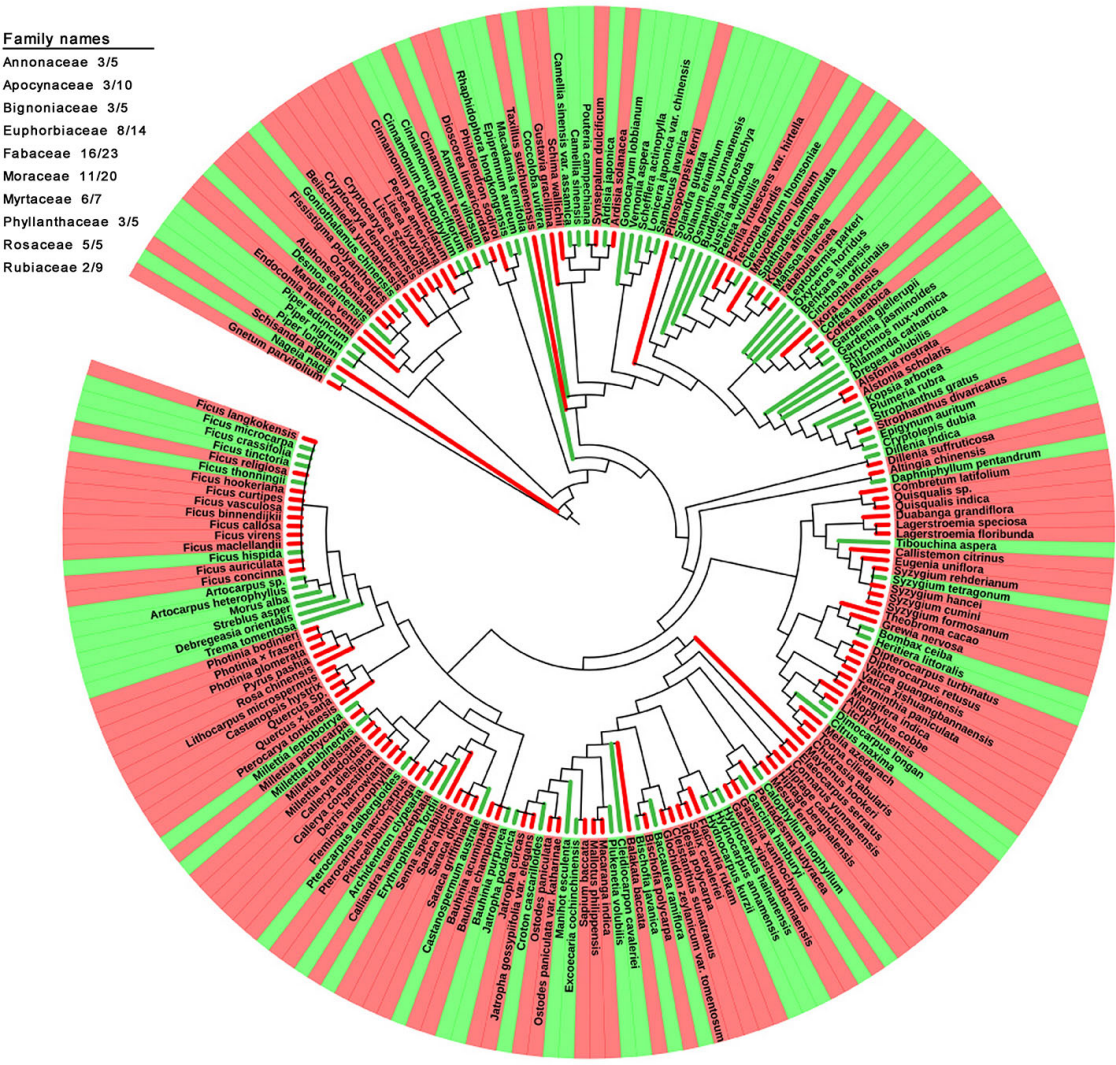
Phylogenetic development tree map of 205 studied plant species with red and green young leaves.

The phylogenetic relationships between the 204 studied species were reconstructed (**Figure 5**). The occurrence of redness in young leaves was not closely associated with their phylogenetic relationships. Plants with young leaves that flush red can be allied closely to other DGL taxa, or can belong to genera with many taxa with different coloration. However, the occurrence of redness in young leaves is a universal phenomenon. For example, 8 out of 14 studied species in the Euphorbiaceae showed young red leaves, 16 out of 23 in the Fabaceae, 11 out of 20 in the Moraceae and 6 out of 7 in the Myrtaceae. Additionally, all studied taxa from the Dipterocarpaceae, Fagaceae, Lythraceae, Myrtaceae, Rosaceae, and Salicaceae exhibited red-colored young leaves. Conversely, we also found that all studied taxa in the Achariaceae and Piperaceae have green young leaves.

## Discussion

The anthocyanin pigments and the tannins, which are defense chemicals, are known to share the same biosynthetic pathway, the phenylpropanoid pathway (Ishikura et al., 1984). It is therefore not surprising that both have a relatively higher accumulation within red young leaves in the present research. Recent literature suggests that high levels of hydrolysable tannins [mainly galloylglucoses/ellagitannins (GGs/ETs)] in the young leaves of plants can inhibit POD mediated anthocyanin degradation and can increase stability and color intensity of anthocyanins by copigmentation activity (Luo et al., 2019). Previous studies have also reported that the delayed greening strategy is widespread in the tropics (Coley and Kursor, 1996; Dominy et al., 2002), and that young tropical leaves with visually striking red coloration have a greater increase in chemical defenses compared with mature leaves than do their temperate counterparts (Coley and Aide, 1989). The ecological function of anthocyanins is hotly debated. Our field observations show that young leaves from DGL plants can more efficiently escape insect herbivory with low leaf damage (17.75 ± 3.30%) compared to young GL leaves (26.89 ± 2.72%, **Figure 1**). We were also able to confirm that young DGL leaves are better able to defend against insect herbivory than are mature leaves. We predict that a higher concentration of anthocyanins and tannins also may plays a role in screening against harmful UV radiation, and can enhance chemical defenses separately based on the generalization of functional ecological specificity.

Theoretically, the low toughness and high nutritive value of young leaves may mean that they are preferred by grazers. Chen and Huang (2013) reported that young red leaves have fewer mechanical defenses than green leaves. Moreover, some studies showed that insect herbivores caused higher leaf area damage to young leaves (Coley and Aide, 1989; Coley and Barone, 1996). These results do not contradict our findings from the Asiatic tropics. Generalist herbivores prefer mature leaves, but they show lower performance than specialist herbivores with specific secondary metabolites (Blüthgen and Metzner, 2007). Therefore, the young DGL leaves may selectively protect themselves through chemical defenses because they have not got physical defense mechanisms.

In the present study, DGL plant taxa produced high amounts of tannins and anthocyanins in those leaves, 13.81 mg TAE/g FW, and 44 A_530nm_/g FW, respectively (**Figure 2**). Tannins have been widely used as an index of plant chemical defense, as they considerably lower the palatability of leaf tissue (Lowman and Box, 1983). They can defend leaves against insect herbivores by deterrence or toxicity. At the same time, tannin-rich leaves contain high concentrations of anthocyanins because these components share the initial steps of the same synthetic pathway (Winkel-Shirley, 2002). Anthocyanins not only contribute to the redness of young leaves but also can effectively scavenge super-oxide and hydrogen peroxide generated by insect-mediated or mechanical damage through the browning reaction (Gould et al., 2002a).

In the tropical wild, plants face not only selection pressure from herbivory, but also abiotic environmental stresses, such as high light exposure, especially in the tropical rainforest zone. Excessive light can cause photo-inhibition, accompanied by damage to the photosynthetic apparatus and a decrease in the photosynthetic rate and can even cause photo-oxidation (Demmig-Adams and Adams, 1992).

A typical plant taxa has an *F_v_*/*F_m_* that ranges from 0.75 to 0.85 (Björkman and Demmig, 1987; Hogewoning et al., 2012). In this study, young DGL leaves had the lowest *F_v_*/*F_m_* ratio (0.70) and all non-red leaves had relatively high *F_v_*/*F_m_* values (0.77 ~ 0.79) (**Figure 3A**). Red leaves exhibited strikingly lower *F_v_*/*F_m_* ratios than did non-red leaves (**Figure 3A**). Thus, our results suggest the occurrence of photo-inhibition to some extent in young DGL leaves.

Plants can dissipate excessive light energy absorbed by light-harvesting complexes through non-photochemical quenching (*NPQ*), which is based on the xanthophyll cycle (Müller-Moulé et al., 2002; Tietz et al., 2017). *NPQ* as measured in this study does not represent the total capacity for non-photochemical quenching of the samples, but it is rather a measure of the instantaneous level of this process depending on the light incident on the sample surface, light absorptivity, energy pressure on the photosystems and photo-protective potential (i.e., xanthophyll pool size). The *Φ_PSII_* calculated was also an instantaneous measurement of the operational efficiency of the *PSII* photochemistry, and as such has little to do with the potential capacity for photo protection. In this study, young DGL leaves exhibited significantly greater *NPQ* levels than mature green leaves from DGL plants, but displayed no significant differences from GL plant species (**Figure 4C**). This suggests that *NPQ* could provide photo protection for red-colored young leaves to some extent. The young DGL leaves exhibited significantly lower *Φ_PSII_* efficiency (0.74) than did GL leaves (**Figure 4B**). This result was closely associated with lower efficiency of light energy utilization, and suggests that the photosynthetic apparatus of young DGL leaves was not damaged by normal photosynthesis and that red leaves can most probably dissipate excess light energy through other mechanisms.

In addition to their role in *NPQ*, anthocyanins may also be able to function as ideal photo-protective agents because of their spectral absorption characteristics and anti-oxidative properties (Hughes et al., 2005; Zhu et al., 2018) although this is still in dispute. *NPQ* and anthocyanins are likely to be two important tools that can provide photo-protective function in plant leaves through different mechanisms. Anthocyanins-mediated photo-protection always occurs at species-specific developmental stages (Zhu et al., 2018). Combined with the high anthocyanin concentrations of young red leaves, anthocyanins may function as light attenuators and ameliorate the effects of excess light energy. However, the lowest *F_v_*/*F_m_* values observed at young DGL leaves in this study showed that photo-inhibition to some extent occurred. Thus, the high concentrations of anthocyanin in young DGL leaves might not have compensated for the insufficient photo-protection mediated by *NPQ* in these leaves but may enhance the fitness of young plants in tropical rainforests against herbivory damage.

## Conclusion

In this interspecific, ecophysiological study, the occurrence of transient reddening of juvenile leaves in the tropics was coupled with increased levels of both anthocyanins and tannins. These two classes of compound were found to co-existed in transiently red young leaves and may have an intrinsic quantitative correlation mediated through POD enzyme activities.

Young leaves that flush red in tropical rainforest plants enhance plant defense and increase plant fitness. Moreover, the occurrence of red coloration in young leaves has arisen many times independently and is present in a variety of unrelated families (**Figure 5**). This indicates that red coloration in young leaves is predominantly a result of adaptation to special tropical environmental conditions but without significant intrinsic phylogenetic relationship of plant species. Our findings suggest that the red coloration of young leaves, which contain high concentrations of tannin and anthocyanin, mainly functions as an anti-herbivore defense strategy with chemical components. Furthermore, anthocyanins, which are principally responsible for the redness of young leaves, might not function as light attenuators to compensate for insufficient photo-protection mediated by *NPQ* because of the lowest *F_v_*/*F_m_* values, but may enhance the fitness of young plants in tropical rainforests to defense herbivory damage.

## Data Availability Statement

All datasets for this study are included in the article/**Supplementary Material**.

## Author Contributions

W-CG and L-ZM planned and designed the research. W-CG, Y-HL, C-MW, Y-QC, and L-ZM collected the data. W-CG and L-ZM contributed to data compilation. W-CG analyzed the data with support from L-ZM and C-M-W. W-CG, KM, and L-ZM wrote the manuscript with contributions from C-MW. All authors gave final approval for publication.

## Conflict of Interest

The authors declare that the research was conducted in the absence of any commercial or financial relationships that could be construed as a potential conflict of interest.

## Supplementary Material

Supplementary Table 1 Name list of plant species used in analysis of photosynthesis physiology and defense chemical content.
